# Structural Basis for Substrate Specificity of Mammalian Neuraminidases

**DOI:** 10.1371/journal.pone.0106320

**Published:** 2014-09-15

**Authors:** Victoria Smutova, Amgad Albohy, Xuefang Pan, Elena Korchagina, Taeko Miyagi, Nicolai Bovin, Christopher W. Cairo, Alexey V. Pshezhetsky

**Affiliations:** 1 Division of Medical Genetics, Sainte-Justine University Hospital Research Center, University of Montreal, Montréal, Canada; 2 Alberta Glycomics Center, Department of Chemistry, University of Alberta, Edmonton, Alberta, Canada; 3 Shemyakin and Ovchinnikov Institute of Bioorganic Chemistry, Moscow, Russia; 4 Institute of Molecular Biomembrane and Glycobiology, Tohoku Pharmaceutical University, Sendai, Miyagi, Japan; University of Toulouse - Laboratoire d'Ingénierie des Systèmes Biologiques et des Procédés, France

## Abstract

The removal of sialic acid (Sia) residues from glycoconjugates in vertebrates is mediated by a family of neuraminidases (sialidases) consisting of Neu1, Neu2, Neu3 and Neu4 enzymes. The enzymes play distinct physiological roles, but their ability to discriminate between the types of linkages connecting Sia and adjacent residues and between the identity and arrangement of the underlying sugars has never been systematically studied. Here we analyzed the specificity of neuraminidases by studying the kinetics of hydrolysis of BODIPY-labeled substrates containing common mammalian sialylated oligosaccharides: 3′Sia-LacNAc, 3′SiaLac, SiaLe_x_, SiaLe_a_, SiaLe_c_, 6′SiaLac, and 6′SiaLacNAc. We found significant differences in substrate specificity of the enzymes towards the substrates containing α2,6-linked Sia, which were readily cleaved by Neu3 and Neu1 but not by Neu4 and Neu2. The presence of a branching 2-Fuc inhibited Neu2 and Neu4, but had almost no effect on Neu1 or Neu3. The nature of the sugar residue at the reducing end, either glucose (Glc) or *N*-acetyl-D-glucosamine (GlcNAc) had only a minor effect on all neuraminidases, whereas core structure (1,3 or 1,4 bond between D-galactose (Gal) and GlcNAc) was found to be important for Neu4 strongly preferring β3 (core 1) to β4 (core 2) isomer. Neu3 and Neu4 were in general more active than Neu1 and Neu2, likely due to their preference for hydrophobic substrates. Neu2 and Neu3 were examined by molecular dynamics to identify favorable substrate orientations in the binding sites and interpret the differences in their specificities. Finally, using knockout mouse models, we confirmed that the substrate specificities observed *in vitro* were recapitulated in enzymes found in mouse brain tissues. Our data for the first time provide evidence for the characteristic substrate preferences of neuraminidases and their ability to discriminate between distinct sialoside targets.

## Introduction

Glycoproteins and glycolipids containing sialic acid (Sia) are found in abundance in mammalian cells, forming a dense array that covers the cell plasma and lysosomal membranes with complex sialylated structures [1] often in a form of dynamic microdomains. The majority of plasma membrane-associated, secreted or lysosomal proteins contain Sia as part of their glycan chains and this modification extends their half-life [2]. In mammals, the content of Sia strongly depends on the cell and tissue type, and changes significantly during development [3].

Due to their diverse physical and chemical properties, Sia are involved in a surprising variety of biological processes [4]. The most important role of Sia is the modulation of recognition events. Sia are well known as commonly exploited ligands for virus, bacteria, and protozoan pathogens. Sia also function as crucial recognition markers in multicellular organisms where they mediate a variety of biological phenomena, including cell differentiation, interaction, migration, adhesion, and metastasis [4]–[6]. Members of the sialic acid binding immunoglobulin-like lectin (Siglec) superfamily mediate intracellular interactions which contribute to the scavenging function of macrophages, pathogen uptake and antigen presentation [7]. Glycosynapses mediate cell signaling and participate in processes such as cell adhesion, motility and growth [8]. Cancer cells have long been recognized to have a significant over-expression of Sia on the cell surface [*e*.*g.* [9]–[12]]. Lipid- and protein-bound Sia are elevated in plasma from cancer patients [10], [13]-[16] and linked with acute phase conditions and chronic disease [*e.g.* [17], [18]].

In mammals, the synthesis of sialoglycoconjugates is performed by a family of 20 sialyltransferases that catalyze the transfer of Sia from cytidine monophosphate-Sia to an acceptor carbohydrate [19], whereas the removal of Sia by enzymatic cleavage is mediated by neuraminidases, represented in vertebrates by four gene families *(NEU1-4)*. These enzymes have different, yet overlapping tissue expression, intracellular localization and substrate specificity. *NEU1* is ubiquitously expressed with the highest levels in kidney, pancreas, skeletal muscle, liver, lungs, placenta and brain [20]. In these tissues, *NEU1* generally shows 10–20 times higher expression than *NEU3* and *NEU4*, and ∼10^3^–10^2^ higher expression than *NEU2* [21]. *NEU3* has the highest expression in adrenal gland, skeletal muscle, heart, testis and thymus [22], [23]. *NEU4* has the highest expression in brain, skeletal muscle, heart, placenta and liver [21], [24], [25]. In the cell, Neu1 is localized at the lysosomal and plasma membranes [26], [27]. Neu2 is a soluble protein found in the cytosol [28]–[31]. Neu3 is a membrane-associated protein localized in the caveolae microdomains of plasma, endosomal and lysosomal membranes [32], [33]. The *NEU4* gene is spliced in 2 different forms differing in the first 12 N-terminal amino acids [21], [34]. The short isoform was found predominantly on the ER membrane [21], [34], whereas the long form is targeted both to mitochondria [21], [35] and lysosomes [24].

Previous studies have defined some biological functions for the four mammalian neuraminidases. Neu1 is crucial for the lysosomal catabolism of sialylated glycoconjugates [36]–[40], but also participates in regulation of cell signaling through desialylation of plasma membrane receptors [41]. In contrast to other neuraminidases, Neu1 is active only in a complex with the lysosomal protease, cathepsin A (CathA) [42]. Genetic deficiency of Neu1 in humans results in a severe metabolic disease, sialidosis (MIM #256550) [43]. In addition, genetic deficiency of *CATHA* results in the secondary deficiencies of Neu1 and lysosomal β-galactosidase and causes the lysosomal storage disorder, galactosialidosis (MIM #256540) [44], [45]. Both disorders manifest clinically with skeletal and gait abnormalities, progressive impaired vision, ataxia, seizures and myoclonus syndrome. The biological role of Neu2 remains unknown, but the enzyme has been linked to the alteration of cytoskeletal functions during myoblast differentiation as well as oncogenesis [46]–[49]. Neu3 is a crucial regulator of transmembrane signaling [50] and is implicated in regulation of neurogenesis, carcinogenesis and apoptosis as well as in insulin signaling [51]. Neu4 participates in lysosomal and mitochondrial ganglioside catabolism [24], [52], [53] and the regulation of neuronal cell differentiation [54].

Although there is a diverse array of biological functions ascribed to the four members of the mammalian neuraminidase family, it is not clear if this is a result of different subcellular or tissue localization or the distinct substrate specificities of each isoenzyme. Previous studies of mammalian neuraminidase substrate specificity have mostly relied on their *in vitro* activities against major classes of sialoglycoconjugates: glycolipids, glycoproteins and free oligosaccharides. It has been reported, in particular, that Neu1 is active primarily against sialylated glycopeptides and oligosaccharides with lower activity against gangliosides; Neu3 requires a hydrophobic aglycone[46], giving it a preference for gangliosides; whereas, Neu2 and Neu4 are active against all types of sialylated glycoconjugates including oligosaccharides, glycoproteins and gangliosides [24], [28], [34]. At the same time, the ability of the enzymes to discriminate between the common Sia linkages known to occur in sialoglycans (α2,3-, α2,6-, and α2,8-) or between the identity and arrangement of the underlying sugars has not been studied systematically.

In the current work we have analyzed the activity and specificity of the four mammalian neuraminidases against a panel of fluorescent 4,4-difluoro-5,7-dimethyl-4-bora-3a,4a-diaza-s-indacene-3-propionic acid (BODIPY)-labeled) substrates containing the most common sialylated oligosaccharides found in mammals: 3′SiaLacNAc (S1), 3′SiaLac (S2), SiaLe_x_ (S3), SiaLe_a_ (S4), SiaLe_c_ (S5), 6′SiaLac (S6), and 6′SiaLacNAc (S7). These studies were combined with molecular modeling to define the structural basis of the enzyme specificities. Our data show for the first time that neuraminidases are capable of discriminating between different sialoglycans, providing crucial insight into their biological functions.

## Experimental Procedures

### Synthesis of BODIPY-labeled sialyloligosaccharides

Sialyloligosaccharides containing ω-amino-spacers were synthesized as described previously [55], [56] and labeled with 4,4-difluoro-5,7-dimethyl-4-bora-3a,4a-diaza-s-indacene-3-propionic acid (succinimidyl ester) - BODIPY FLSE (Molecular Probes) [57]. BODIPY-labeled sialyloligosaccharide neuraminidase substrates had the following structures: Neu5Acα2-3Galβ1-4GlcNAc-BODIPY (S1; SiaLe_c_); Neu5Acα2-3Galβ1-3GlcNAc-BODIPY (S2; 3′SiaLac); Neu5Acα2-3Galβ1-4Glc-BODIPY (S3; SiaLe_a_); Neu5Acα2-6Galβ1-4GlcNAc-BODIPY (S4; SiaLe_x_); Neu5Acα2-6Galβ1-4Glc-BODIPY (S5; 6′SialLacNAc); Neu5Acα2-3Galβ1-4(Fucα1-3)GlcNAc-sp-BODIPY (S6; 6′SiaLac); and Neu5Acα2-3Galβ1-3(Fucα1-4)GlcNAc-sp-BODIPY (S7; 3′SiaLacNAc).

### Neuraminidases

Neu1 was purified from mouse kidney tissue by affinity chromatography on a concanavalin A-Sepharose column followed by fast protein liquid chromatography gel filtration on Superose-6 column, as previously described [58]. Neu2 and Neu3 were expressed in *E. coli* fused at N-terminal with maltose-binding protein and Neu4 as a glutathione S-transferase-fusion protein and purified as described [47]–[49]. Neu2 and Neu4 carrying a streptavidin binding peptide tag at the C-terminus were also expressed in COS 7 cells and purified to electrophoretic homogeneity by affinity chromatography on Streptavidin agarose essentially as described [41], [59]. Specific enzymatic activity of purified enzymes was measured against 4-Methylumbelliferyl α-D-*N*-acetylneuraminic acid (4MU-NANA) in 0.1 M Na-acetate buffer at the enzyme optimal pH (4.5 for Neu1, Neu3, and Neu4; and 5.5 for Neu2) and molecular mass was confirmed using FPLC size exclusion chromatography as described [58]. For the MS/MS analysis after separation by SDS-PAGE the gel pieces containing Neu2 and Neu4 were excised, and digested with trypsin (Promega). The tryptic peptides were analyzed by nano-liquid chromatography mass spectrometry (nanoLC-MS) using an Eksigent nano-LC LTQ-Orbitrap mass spectrometer system (Thermo Fisher Scientific) as described [60]. Assignments of phosphorylation sites were validated through manual inspection of relevant MS/MS spectra.

### Neuraminidase activity assay

Neuraminidase activity against BODIPY-labeled substrates was assayed as described by Mochalova et al. [57] with minor modifications. Briefly, the 15 µL of reaction mixture containing 8 mU (1 mU equals 1 nmol of 4MU-NANA substrate converted per h) of neuraminidase enzyme and a substrate at a final concentration of 20 µM, 10 µM, 5 µM, 2.5 µM, 1.2 µM or 0.6 µM in 0.1 M Na-acetate buffer, pH 4.5 for Neu1, Neu3, Neu4 or pH 5.5 for Neu2 was incubated at 37°C for 20 min in 96-well PCR plates (BioScience Inc.). For blank samples, neuraminidase was replaced with an equal volume of water. The reaction was terminated by the addition of 85 µL of ice-cold water and 10-µL aliquots of the reaction mixtures were applied to 96-well filter plates (Millipore, 40 µm Nylon Mesh) containing 40 µL of DEAE-Toyopearl 650 M (Tosoh) resin per well. Prior to the assay, the resin was washed twice with 250 µL of water/well and the plates centrifuged at 50 *g* for 30 s to remove any excess water. After application of the reaction mixture, the DEAE-plates, were incubated for 1 min at room temperature and centrifuged at 50 *g* for 30 sec. Reaction products were eluted with two 95-µL aliquots of water by centrifugation of the plates at 50 *g* for 30 s. Combined eluent (200 µL) was transferred to 96-well ReaderBlack polystyrene plates (Life Science) and the amount of fluorescent product was measured using an EnVision 2104 Multilabel fluorimeter (Perkin Elmer) at emission wavelength of 535 nm and excitation wavelength of 485 nm. Three independent duplicate measurements were performed for each experimental condition. Kinetic parameters of enzymatic reactions were analyzed by non-linear regression using Prism Graphpad software.

### Animals

Mice with targeted disruption of the *neu*3 (*neu*3^−/−^) and *neu*4 (*neu*4^−/−^) genes and hypomorph mice with deficiency of CathA expression causing secondary 90% reduction of the Neu1 activity in tissues (*CathA^S190A-neo^*), all in C57BL/6NCrl genetic backgrounds have been previously described [61]–[63]. Mice with a combined deficiency of Neu4 and Neu3 were obtained by intercrossing *neu4* and *neu3* knockout (KO) mouse strains. Doubly homozygous *neu4* ^−/−^; *neu3* ^−/−^ progeny were viable and their genotypes were confirmed by PCR of tail DNA. The absence of *Neu4* transcripts in total mRNA extracted from the brain of *neu4^−/−^; neu3^−/−^* mice was confirmed by RT-PCR (Figure S1). Mice were housed in an enriched environment with continuous access to food and water, under constant temperature and humidity, on a 12 h light∶dark cycle. Approval for the animal care and the use in the experiments was granted by the Animal Care and Use Committee of the Ste-Justine Hospital Research Center.

### Neuraminidase assay in mouse brain tissues

At the age of 12 weeks mice were sacrificed using CO_2_ chamber and their brains extracted, snap-frozen with liquid nitrogen and kept at −80°C. For measurement of neuraminidase activity 100 mg of frozen brain tissue was homogenized in water in a ratio of 100 mg of tissue per 300 µL of water in the 1.5 mL Eppendorf tubes using Kontes Pellet motorized pestle. Protein concentration in the homogenate was measured by Bradford method using the Bio-Rad reagent. The assay was performed as described above for recombinant neuraminidases but the reaction mixture contained an aliquot of homogenate corresponding to 150 µg of total protein and substrate in a final concentration of 10 µM. The reaction was carried on at 37 °C for 2 h after which it was terminated by the addition of 180 µL of ice-cold water. For blank samples, the reaction mixture contained buffer and substrate only but the same volume of homogenate was added after the termination of reaction with water.

### Molecular modeling

The structures of the substrates were generated using GLYCAM Web online utilities (*GLYCAM Web*, Complex Carbohydrate Research Center, University of Georgia, Athens, GA, http://www.glycam.com). A methyl group was used as the aglycon to simplify calculations. The substrates were docked in the active site of either a previously reported Neu3 homology model [47] or a reported Neu2 crystal structure [54]. Docking calculations were performed with Autodock 4.0, and a grid box of 60*60*60 points centered at *C*2 of the Neu5Ac or 2,3-dehydro-2-deoxy-*N*-acetylneuraminic acid in the enzyme complex. Two hundred docked poses were generated and clustered, the lowest energy clusters maintaining key interactions (e.g. contact with the arginine triad and the *C*1 carboxylate) were selected for further study by molecular dynamics. Topology and coordinate files were generated for calculations in AMBER using the tLEaP module with solvation to generate a neutralized complex. An octahedral box of water (TIP3P) at 7 Å around the complex was used, with Na^+^ ions added to neutralize the complex. Molecular dynamics simulations were performed using the AMBER 10 package. The GLYCAM06 force field was used with the carbohydrate substrates, and the AMBER ff99SB force field was used for the rest of the complex [50].

The complex was prepared for a production run of molecular dynamics by minimization and equilibration. The solvent molecules were first minimized while keeping the substrate constrained. This was followed by a minimization of the entire system. In both minimization steps, a steepest descent energy minimization was carried out for 50 cycles followed by 4950 cycles of conjugate gradient minimization. For equilibration, a total of 600 ps of annealing was used during which the temperature was increased every 50 ps from 5 to 300 K and then cooled back from 300 to 5 K. This was followed by a short equilibration run of 200 ps during which the temperature of the system was gradually increased from 5 K to 300 K over 150 ps, followed by a constant temperature of 300 K for 50 ps. The production simulation (10 ns) was performed under constant pressure and temperature (NPT) with periodic boundaries. The temperature was kept at 300 K and the pressure at 1 atmosphere. The particle mesh Ewald (PME) method and SHAKE algorithm were used. Longer simulations (up to 20 ns) yielded substantially similar results. Simulations were analyzed for convergence by examining the total energy and potential energy of the run. Structures from the equilibrated phase of the production run were used for analysis.

## Results

### Substrate specificity of neuraminidases

**Figure 1:**
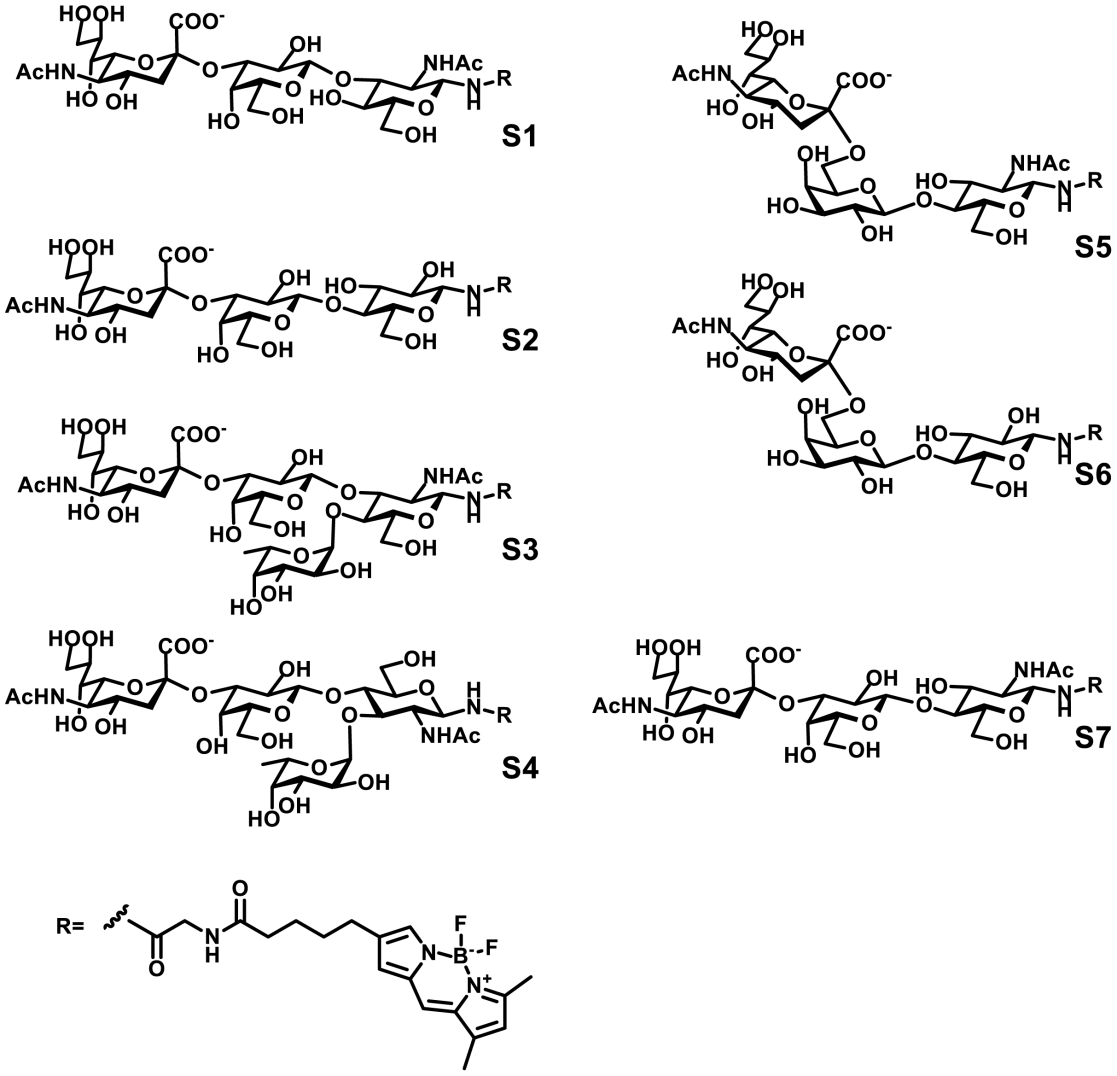
Chemical structures of the fluorescent substrates used in this study.

Substrate specificity of the four mammalian neuraminidases was studied against a panel of synthetic sialo-oligosaccharides, typical for mammalian carbohydrate chains (Figure 1). The oligosaccharides differed in: (1) the type of linkage between Neu5Ac and Gal residues (α2,3 or α2,6; i.e. 3′SiaLac (S2) and 3′SiaLacNAc (S7) vs. 6′SiaLac (S6) and 6′SiaLacNAc (S5), respectively); (2) the presence or absence of the *N*-acetyl group at position two of the Glc residue (i.e. 3′SiaLacNAc (S7) vs. 3′SiaLac (S2)); (3) the linkage (1,4 or 1,3) between Gal and GlcNAc residues (i.e. 3′SiaLacNAc (S7) vs. SiaLe_c_ (S1)); and (4) the presence or absence of a branching Fuc residue at GlcNAc (i.e. SiaLe_a_ (S3) and SiaLe_x_ (S4) vs. SiaLec (S1) and 3′SiaLacNAc (S7) respectively).

The activities against the above substrates were tested with recombinant human neuraminidases 2, 3 and 4 expressed in *E. coli* and purified as previously described [47]–[49]. As we demonstrated previously, recombinant enzymes have specific activities against the fluorogenic substrate, 4MU-NANA and the pH optima similar to those of the enzymes purified from the mammalian tissues [49]. However, it is possible that the mammalian neuraminidases expressed in bacteria, which lack post-translational modifications or an oligomeric structure characteristic for mammalian enzymes, may have different enzymatic properties. To test this we also expressed Neu2 and Neu4 with a C-terminal TAP (SBP/CBP) tag in mammalian COS 7 cells and purified them using affinity chromatography on Streptavidin-agarose. Analysis of the purified enzyme by tandem mass spectrometry demonstrated that both Neu2 and Neu4 contained O-linked phosphorylations and according to FPLC size exclusion chromatography Neu4 expressed in COS 7 cells formed homodimers with an apparent mass of ∼120-kDa. Despite these differences with recombinant enzymes expressed in bacteria, we observed identical profiles of activities for the Neu2 and Neu4 proteins expressed in bacteria and mammalian cells against all substrates (data not shown). Based on these data, all further studies were performed using the recombinant enzymes.

Since Neu1 is catalytically active only as a part of a multienzyme lysosomal complex with β-galactosidase and lysosomal carboxypeptidase, cathepsin A, we used the endogenous enzyme purified from mouse kidney tissue by affinity chromatography on concanavalin A-Sepharose followed by FPLC size exclusion chromatography as previously described [58]. Mouse and human Neu1 have a very high (83% identity, 89% similarity) amino acid homology and similar specificity [42].

**Figure 2:**
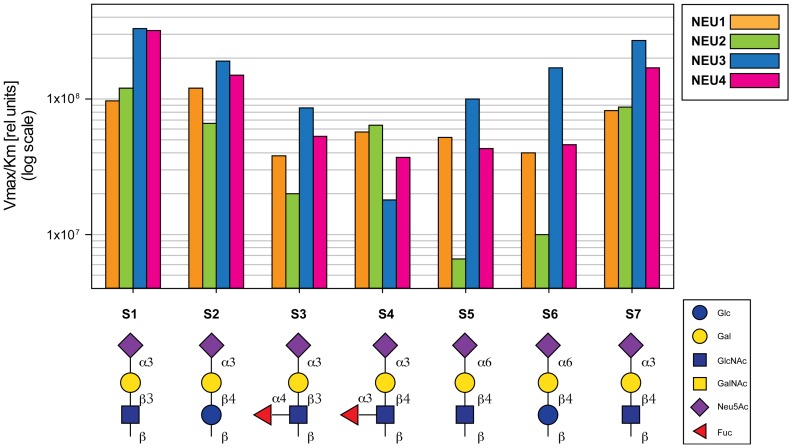
Substrate specificity of mammalian neuraminidases. V_max_/K_M_ values are plotted on a logarithmic scale. Values shown are means of three independent experiments.

For each enzyme the initial reaction rate was measured at a corresponding pH-optimum (4.75 for Neu1, Neu3 and Neu4 and 5.5 for Neu2) for six substrate concentrations between 0.6 and 20 µM. The same amount (8 mU) of the enzyme activity was added to the reaction mixture for each neuraminidase. After a 30-min incubation at 37 °C, the reaction was terminated by dilution with ice-cold water; the product was separated from unreacted substrate using ion-exchange chromatography on Toyoperl DEAE-650 and its concentration was measured using a spectrofluorimeter. In separate experiments we confirmed that under the conditions used the amount of liberated product was directly proportional both to the incubation time and to the amount of enzyme in the reaction mixture (data not shown). For each enzyme the dependence of the initial reaction rate on the substrate concentration could be described by the Michaelis-Menten equation allowing determination of *K*_M_ (Michaelis constant) and V_max_ (maximal velocity of enzymatic hydrolysis) values (Table 1). V_max_/K_M_ ratios were used as a measure of specificity of neuraminidases towards corresponding the substrates (Figure 2).

**Table 1:** Kinetic data from substrate studies with recombinant neuraminidases.

	S1	S2	S3	S4	S5	S6	S7
Neu1							
V_max_ (SE)	3·10^7^ (4.4·10^6^)	2.8·10^7^ (2.4·10^6^)	5·10^25^ (6.9·10^24^)	2.4·10^7^ (9.9·10^6^)	1.4·10^7^ (1.8·10^6^)	2·10^7^ (4.2·10^6^)	3.2·10^7^ (4.5·10^6^)
K_M_ (SE)	0.31 (0.07)	0.24 (0.04)	1.3·10^18^ (1.3·10^17^)	0.42 (0.26)	0.27 (0.06)	0.50 (0.14)	0.39 (0.08)
V_max_/K_M_	9.7·10^7^	1.2·10^8^	3.8·10^7^	5.7·10^7^	5.2·10^7^	4·10^7^	8.2·10^7^
Neu2							
V_max_ (SE)	1.7·10^7^ (1.2·10^6^)	2.5·10^7^ (3.7·10^6^)	1·10^26^ (1.5·10^25^)	2.7·10^7^ (2.3·10^6^)	9.9·10^20^ (1.3·10^20^)	9.3·10^23^ (7.3·10^23^)	2·10^7^ (9.2·10^5^)
K_M_ (SE)	0.14 (0.02)	0.38 (0.08)	5·10^18^ (6.2·10^17^)	0.42 (0.05)	1.5·10^14^ (2.5·10^13^)	8.9·10^16^ (1.2·10^16^)	0.23 (0.02)
V_max_/K_M_	1.2·10^8^	6.6·10^7^	2·10^7^	6.4·10^7^	6.6·10^6^	1·10^7^	8.7·10^7^
Neu3							
V_max_ (SE)	2·10^7^ (1.7·10^6^)	2.7·10^7^ (2.9·10^6^)	4.4·10^7^ (4.2·10^6^)	2.3·10^7^ (2.6·10^6^)	2.8·10^7^ (3·10^6^)	2.2·10^7^ (2.2·10^6^)	1.6·10^7^ (1.6·10^6^)
K_M_ (SE)	0.06 (0.01)	0.14 (0.03)	0.51 (0.07)	0.13 (0.03)	0.23 (0.04)	0.13 (0.03)	0.06 (0.02)
V_max_/K_M_	3.3·10^8^	1.9·10^8^	8.6·10^7^	1.8·10^7^	1.0 ·10^8^	1.7·10^8^	2.7·10^8^
Neu4							
V_max_ (SE)	1.6·10^7^ (1.4·106)	3·10^7^ (5.4·10)^6^	4.5·10^7^ (1.7·10)^7^	4.4·10^7^ (7.4·10^6^)	5.2·10^6^ (1.3·10^6^)	1.1·10^7^ (3.6·10^6^)	1.9·10^7^ (2.8·10^6^)
K_M_ (SE)	0.05 (0.01)	0.20 (0.07)	0.85 (0.42)	1.18 (0.83)	0.12 (0.06)	0.24 (0.14)	0.11 (0.04)
V_max_/K_M_	3.2·10^8^	1.5·10^8^	5.3·10^7^	3.7·10^7^	4.3·10^7^	4.6·10^7^	1.7·10^8^

Values shown are means and standard errors of three independent experiments.

In general, Neu3 and Neu4 were more active than Neu1 and Neu2 against most substrates in the panel. Importantly, neuraminidases also showed drastic differences in specificity towards substrates with 2,6-linked and 2,3-linked Sia. Neu3 showed similar activity for an α2,6-linked-substrate, 6′SiaLac (S6), and the α2,3-linked isomer, 3′SiaLac (S2). The specificity of Neu1 was similar for the 6′SiaLacNAc (S5) and 3′SiaLacNAc (S7). Neu4 was capable of hydrolyzing α2,6-linked Sia substrates, but showed more than 3-fold-reduced specificity for 6′SiaLac (S6) and 6′SiaLacNAc (S5) as compared to 3′SiaLac (S2) and 3′SiaLacNAc (S7). Neu2 showed virtually no activity against substrates with α2,6-linked Sia (S5 and S6).

The nature of the residue at the reducing end (Glc or GlcNAc) had only a minor effect on the activity of all neuraminidases, with a similar specificity observed for 3′SiaLac (S2) and 3′SiaLacNAc (S7) and for 6′SiaLac (S6) and 6′SiaLacNAc (S5). The central Gal-GlcNAc linkage (β1,3 or β1,4) had a noticeable effect on Neu4 activity, which showed 2-fold-reduced specificity for the β1,4-linked GlcNAc substrate (S1 vs. S7).

The branching Fuc residue generally reduced substrate activity for all neuraminidases. This effect was more pronounced when Fuc was attached in α1,4 position to GlcNAc residue (S3 vs. S1) rather than in α1,3 position (S4 vs. S7). Of all enzymes Neu4 was the most affected by the branching Fuc, which reduced the specificity for the corresponding substrates as much as ∼6-fold. Neu1 was least affected by fucosylation, with only 2-fold reduction of specificity for branching Fuc attached both in α1,4 and α1,3 positions.

### Molecular modeling of substrate binding in the active sites of Neu2 and Neu3

To interpret the differences in the specificities of the enzymes we used molecular modeling to dock specific substrates to the active site of interest. There are limited structural data available for the mammalian neuraminidases with structures reported only for Neu2. Although homology models of the remaining three isoenzymes have been described, only Neu3 has been experimentally tested [47]. We restricted our modeling studies to the Neu2 and Neu3 active sites for which we identified sets of substrates with large differences in the V_max_/K_m_ ratio.

Models of enzyme-substrate complexes were generated using molecular dynamics. Initial protein structures were based on crystallographic data in the case of Neu2 [54], and a homology model in the case of Neu3 [47]. Substrates were first docked into the active site using Autodock 4, and then subjected to molecular dynamics to convergence, followed by minimization as described in Materials and Methods (Table 2).

**Table 2:** Predicted protein-ligand contacts in Neu2 and Neu3.

		S1	S5	S1
		Neu2	Neu2	Neu3
monosaccharide	site	residue	residue	residue
Neu5Ac	C1-COOH	R304 R237 Y334	R304 R21	R340 R25 Y370
	4-OH	R41	R41	R45
	5-NH	Y334		E225 Y370
	5-C(O)	R41		R45
	7-OH	E111		R245
	8-OH	E111	E111	D50
	9-OH	R237	E218 R237	Y179
Gal	2-OH		K44*	
	3-OH		Y359	
	4-OH			
	6-OH			
GlcNAc	2-NH			
	3-OH		Q112	
	4-OH	E111		R245
	5-O			
	6-OH	D46Q112	D46	

*Backbone interactions (carbonyl or amide).

**Figure 3:**
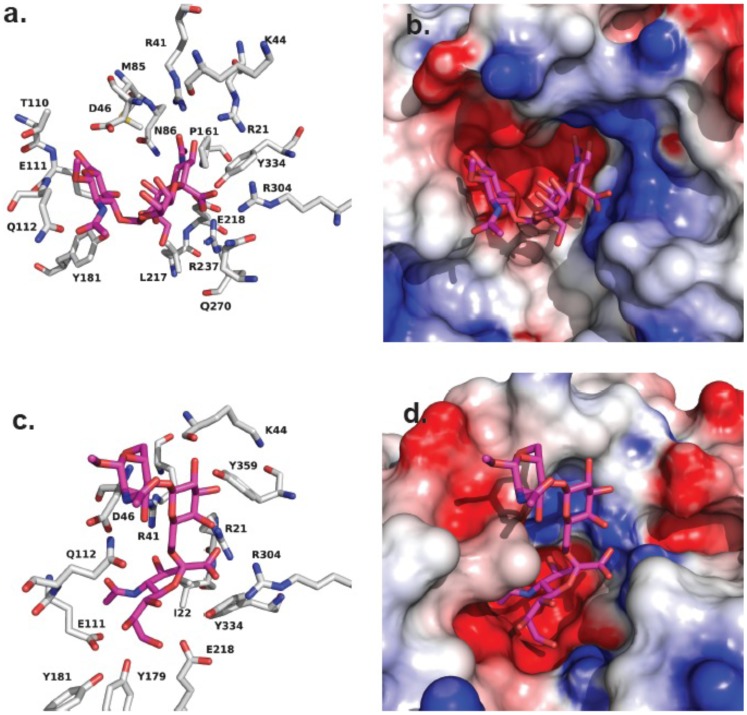
Modeling of S1 and S5 binding to the Neu2 active site. (a) The substrate, S1, binds to the active site with expected contacts to the arginine triad and glycerol side chain. The GlcNAc residue B face is placed on the top of the hydrophobic side chain of Q112, forming a favorable interaction. The α2,3-glycosidic linkage between Neu5Ac and Gal allows S1 to form a relatively stable complex. (b) The same position for S1 as shown in (a), but with a surface representation of the Neu2 active site. (c) The α2,6-glycosidic linkage between the Neu5Ac and Gal residues in S5 makes it difficult for the substrate to fit into the active site without adopting an unfavorable conformation. The Neu5Ac residue adopts a twist boat conformation to preserve interactions with the Arg triad. The GlcNAc A face is interacting with a hydrophobic patch in the Neu2 active site, which is likely unfavorable. (d) The same pose for S5 as shown in (c), but with a surface representation of the Neu2 active site.

Neu2 showed the largest differences in activity against substrates S1 (SiaLe_c_) and S5 (6′SiaLacNAc). The two substrates are isomers differing in the two glycosidic linkages of Sia-Gal (α2,3 vs. α2,6) and the Gal-GlcNAc (β1,3 vs. β1,4) residues. The model of S1 binding to the active site of Neu2 includes most of the expected key interactions seen in co-crystals with sialosides (Figure 3) [54], including binding of the *C*1-carboxylate of Sia with the Arg triad. The GlcNAc residue B face is placed on the top of the hydrophobic side chain of Glu112 forming favourable non-polar contacts. Thus, the α2,3 glycosidic linkage between Sia and Gal residues allows the S1 substrate to form the stable complex with the active site of the enzyme. In contrast, the α2,6 glycosidic linkage of S5 makes it difficult for the substrate to fit into the Neu2 active site without adopting an unfavorable conformation. The best orientation of S5 in the Neu2 active site results in Sia adopting a twist-boat conformation in order to preserve interactions of the Arg triad and the *C*1-carboxylate. The GlcNAc A face of S5 develops an interaction with a hydrophobic patch of the Neu2 surface (C_b_, D46). This model suggests that the reduced activity of S6 may also be a result of the α2,6 linkage forcing an unfavorable orientation of the Sia residue. Considering that S7, which also contains a β1,4 glycosidic linkage to GlcNAc, has activity comparable to S1 with Neu2; we conclude that the glycosidic linkage of Sia is the major determinant of substrate activity in this case.

**Figure 4:**
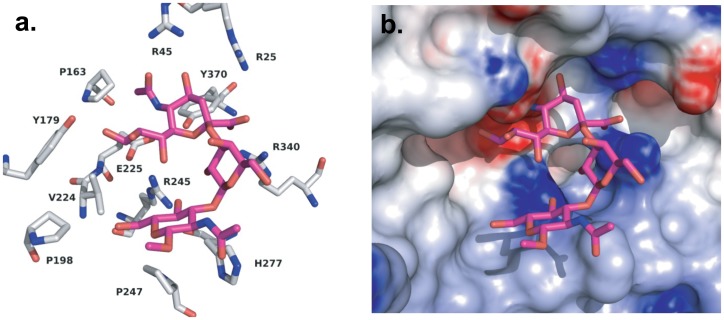
Modeling of S1 binding to Neu3 active site. (a) Interactions of S1 with amino acid residues in the active site of the Neu3 homology model. (b) The same position of S1 shown in (a), with a surface representation of the active site of Neu3.

We next examined the interaction of the most active (S1; SiaLe_c_) and least active (S3; SiaLe_a_) substrates with the active site of Neu3. The only structural difference between the two substrates is the branching Fuc1,4 residue of S3. A model of S1 binding to the active site of Neu3 was generated using the procedure described in Materials and Methods (Figure 4). In this model, the Neu5Ac residue adopts a twist boat conformation in order to preserve many key interactions [64]. However, when we attempted the same procedure for S3, we were unable to identify any suitable orientation for the substrate that would place the Sia residue in the correct orientation. Upon inspection of the S1-Neu3 model, we concluded that the branching fucosyl residue of S3 would be directed deeper into the binding site where it would clash with protein side chains (R245 and E225). Thus, we concluded that branching at *O*4 of GlcNAc prevented appropriate binding of the substrate. This would likely be less of an issue for compounds like S4, which could accommodate the α1,4-fucosyl residue by projection into solvent when the Gal moiety is linked in β1,3 position.

### Neuraminidase activity in the mouse brain tissues

In order to determine if the detected differences in the specificities of neuraminidases against the BODIPY-labeled sialylated oligosaccharides could be used to discriminate between the enzymes *in vivo*, we have assayed neuraminidase activity in the tissues of available previously described gene-targeted mouse models deficient in Neu1 (*CathA^S190A-neo^*, *neu1 KI*), Neu3 (*neu3^−/−^*) and Neu4 (*neu4^−/−^*)[61] [62], [63]. As previous reports have suggested that Neu3 and Neu4 have similar substrate specificity [21], [24] we also produced a mouse line with a double Neu3/Neu4 deficiency (*neu3^−/−^; neu4^−/−^*) by cross-breeding individual knockouts. As expected the expression levels of both *neu3* and *neu4* were below the detection limit in the tissues of the *neu3^−/−^; neu4^−/−^* mice (Figure S1).

**Figure 5:**
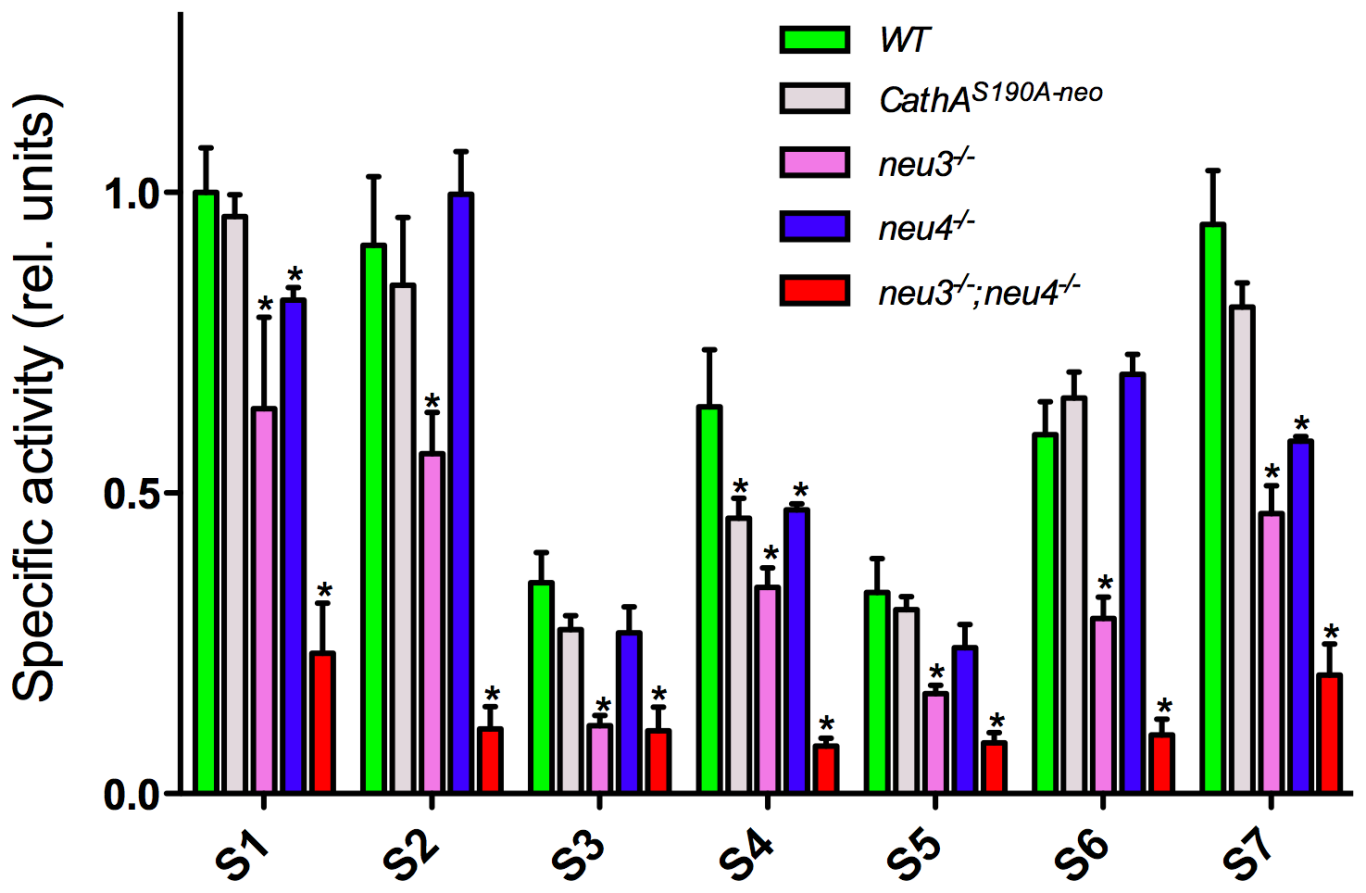
Neuraminidase activity in mouse brain tissues. The activity was measured in the homogenates of whole brain tissues of wild-type C57Bl6 mice (*WT*) and gene-targeted C57Bl6 mice deficient in Neu3 (*neu3* ^−/−^), Neu4 (*neu4* ^−/−^), Neu1 (*CathA^S190A-neo^*) and double-knockout *neu3* ^−/−^; *neu4* ^−/−^ mice against BODIPY-labeled sialylated oligosaccharides in 10 µM concentration. Values are shown as means (±S.E). N-value for each genotype is as follows: for WT and *neu3* ^−/−^; *neu4* ^−/−^ n = 8, for *neu3* ^−/−^ and *neu4* ^−/−^ n = 6; for *CathA^S190A-neo^* n = 4. * -significantly different from WT (P<0.05) by repeated measurements ANOVA.

We assayed the neuraminidase activity in the mouse brain tissues, where approximately equal amounts of Neu1, Neu3 and Neu4 (and only negligible amount of Neu2) were previously found in the WT mice[65]. Acidic neuraminidase activity, assayed using 4MU-NaNa as substrate, was reduced to 38% of the WT level in the brain tissues of *neu1 KI* mouse, 64% in the *neu3^−/−^* mouse, 59% in the *neu4^−/−^* mouse, and 36% in the *neu3^−/−^; neu4^−/−^* mouse (Figure S2), indicating that each of the three neuraminidases (Neu1, Neu3 and Neu4) contribute approximately 30% of the net brain neuraminidase activity against 4MU-NaNa. In contrast, the residual neuraminidase activity levels measured in the brain tissues of Neu-deficient mice against BODIPY-labeled sialylated oligosaccharides were drastically different between substrates (Figure 5). First, we did not detect a significant reduction of the activity for any substrate except of the substrate S4 (SiaLe_x_) in the tissues of Neu1-deficient mice as compared to that in the WT mice. This is not surprising because S4 is the only substrate for which Neu1 showed higher specificity then Neu3 or Neu4 (Figure 2), whereas the rest of the substrates were hydrolyzed by Neu3 and Neu4 with the rates at least two fold higher than that of Neu1. Second, the activity in the tissues of both n*eu3* and *neu4* KO mice against the substrates S1, S4, and S7 (equally specific in vitro for Neu3 and Neu4) was significantly lower than that in the tissues of WT mice (Figure 5). This was consistent with the data on the residual activity against S1, S4, and S7 in double-knockout *neu3^−/−^; neu4^−/−^* mice which was significantly lower than that in *neu3* or *neu4* knockouts. On the other hand, the activity measured against the Neu3-specific substrates S3, S5 and S6 was similar in the tissues of *neu4* KO and WT mice but was equally reduced in the tissues of *neu3* KO and double-KO *neu3^−/−^; neu4^−/−^* mice. These substrates can be potentially used for specific measurements of Neu3 activity in tissue homogenates. Finally, although *in vitro* Neu3 and Neu4 were equally specific for S2 the activity against this substrate in the tissues of *neu4* knockout mice was not reduced as compared with WT mice. In the *neu3* KO mice the activity measured against S2 was lower than in WT but higher than in the double-KO *neu3^−/−^; neu4^−/−^* mice, which may imply that changes in the *neu3* expression may help to compensate for the deletion of the *neu4* gene product, consistent with a significant increase of the *neu3* mRNA in the brain tissues of *neu4^−/−^* as compared to that in WT mice (Figure S1).

## Discussion

Phylogenetic analysis of the sialidase/neuraminidase family suggests that it has originated in bacteria, and then specialized forms were developed in higher organisms [66]. Neu1 was likely the first to form, further evolving into Neu2 and a common precursor of Neu3 and Neu4. The development of members of the neuraminidase family other than Neu1 was most likely driven by a classical mechanism of subfunctionalization, where different isoforms would have distinct biological functions and substrate specificity [66].

Our analysis shows that Neu1, the oldest member of the neuraminidase family, also has the broadest specificity. The Neu1 enzyme is active against both α2,3 and α2,6 linked Sia. Its activity is not affected by the core type (1,3 or 1,4 bond between Gal and GlcNAc residues) and is only slightly inhibited by the branching Fuc residues. Such broad substrate specificity explains the wide diversity of the biological substrates and functions of Neu1. First, as a lysosomal enzyme Neu1 catabolizes a wide range of glycan chains on sialylated glycoproteins, which contain both α2,3 and α2,6 linked Sia and are often fucosylated [42]. The role of Neu1 in the lysosomal catabolism of gangliosides is still disputed. The enzyme has much lower in vitro activity against different gangliosides as compared with Neu3 and Neu4, which could be compensated by the high intra-lysosomal content of Neu1. The data on storage of gangliosides in human sialidosis patients have been controversial [reviewed in [42]]. Our current data show that Neu1 is capable of cleaving Sia residues from glycans containing both GlcNAc and Glc in the third position, and present in di-sialo and mono-sialo gangliosides, respectively. Besides catabolism, Neu1 has important regulatory functions, such as desialylation of multiple surface receptors in immune, metabolic, and cell proliferation pathways [reviewed in [67]]. The broad specificity of Neu1 we report here helps to explain the enzyme's ability to act on a wide range of receptors with diverse structures of the glycan chains.

Of all neuraminidases, Neu2 has the narrowest specificity. Interestingly, the enzyme also has a very restricted expression pattern: it was cloned from skeletal muscle [28], [68] and thymus [69] tissues, but placenta, testis, ovary and lungs have been recently identified as its major expression sites in humans. The enzyme has been proposed to cleave the G_M3_ ganglioside in differentiating myoblasts [70], [71], but no direct evidence has been reported. In PC-3 prostate cancer and melanoma cells, Neu2 activity has been correlated with invasive and metastastatic potential [65]; however, the biological substrates of Neu2 involved in this process remain to be identified. Our data confirm previous observations that Neu2 has a preference for α2,3-linked Sia[72] but also show that Neu2 is active on glycan chains lacking a branching Fuc residue.

The Neu3 and Neu4 isoenzymes show highest phylogenetic similarity of all mammalian neuraminidases [66], and have relatively similar profiles of substrate specificity. Both enzymes have higher activity when compared to Neu1 and Neu2 against all substrates from our panel with an exception of S4. This observation could be the result of the fact that all substrates contain a bulky hydrophobic aglycone (BODIPY). Previous studies have found that Neu3 prefers substrates containing a hydrophobic aglycone [46] presumably interacting with a hydrophobic binding site in Neu3 containing V222, V224, P198, P247, I117, and V118 residues of the enzyme (A. Albohy, M.R. Richards, and C.W. Cairo, unpublished). Additionally, both Neu3 and Neu4 are known to cleave ganglioside substrates in vitro, suggesting that gangliosides are their main physiological substrates [24], [52], [73]. For both Neu3 and Neu4 the most active substrate is S1 containing α2,3 linked Sia and β1,3 linked GlcNAc at the third position, followed by S7 containing β1,3 linked GlcNAc. Neu4 has previously been shown to cleave SiaLe_x_ and SiaLe_a_ structures, and our data confirm that the equivalent glycans (S4 and S3) are substrates of this enzyme [74]. The only apparent difference in specificity between Neu3 and Neu4 is their ability to cleave α2,6 linked Sia. While Neu4 showed reduced activity towards substrates containing α2,6 linked Sia, Neu3 showed only a slight reduction. Similarly the activity of Neu3 was less affected by branching Fuc residues. Since α2,6 linked Sia are found preferentially in glycans of glycoproteins, we speculate that membrane glycoproteins may be among the physiological substrates of Neu3, which is in contrast to the common notion that Neu3 is a purely ganglioside-specific neuraminidase.

In conclusion, this study is the first systematic analysis of the substrate specificity of mammalian neuraminidases at the level of glycan structures. Our data reveal significant differences in the substrate specificity between the four mammalian neuraminidases presumably resulting from the structural organization of their active sites and suggest that presence of four different genes encoding enzymes responsible for removal of Sia residues from glycoconjugates is explained by the need for different substrate specificities. The described substrate preferences for each neuraminidase may be crucial therefore for future identification of their biological targets and pathways involving these enzymes. Besides, our results on measurement of neuraminidase activity in mouse models deficient in individual neuraminidases show that some of described substrates or their analogues can be potentially used to measure activities of the specific sialidases *in vivo*.

## Supporting Information

Figure S1 **Relative expression of neuraminidase mRNA in mouse brain tissues.** Total mRNA was extracted from whole brains of 16 week-old WT, *neu3* ^−/−^, *neu4* ^−/−^ and double-knockout *neu3* ^−/−^; *neu4* ^−/−^ mice and analyzed for *neu1, neu2*, *neu3* and *neu4* expression by qRT-PCR The values were corrected for the level of control *RPL32* mRNA. ** and *** -significantly different from WT (P<0.01 and P<0.001, respectively) by repeated measurements ANOVA.
(PDF)

Figure S2 **4MU-NANA neuraminidase activity in mouse brain tissues.** Brain tissues of 16 week-old WT, *neu3* ^−/−^, *neu4* ^−/−^, *CathA^S190A-neo^* (*neu1 KI*) and double-knockout *neu3* ^−/−^; *neu4* ^−/−^ mice and analyzed for neuraminidase activity against 4MU-NANA. Values are shown as means (±S.E). N-value for each genotype is as follows: WT and *neu3* ^−/−^; *neu4* ^−/−^ n = 8, *neu3* ^−/−^ and *neu4* ^−/−^ n = 6; *neu1 KI* n = 4. *** -significantly different from WT (P<0.001) by repeated measurements ANOVA.
(PDF)
