# Supplementary figures and images for: Structural Basis for Substrate Specificity of Mammalian Neuraminidases
Source: PLoS One. 2014 Sep 15;9(9):e106320. doi: 10.1371/journal.pone.0106320 (PMC4164519; doi:10.1371/journal.pone.0106320)

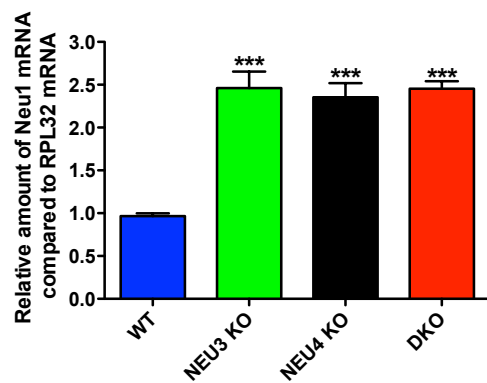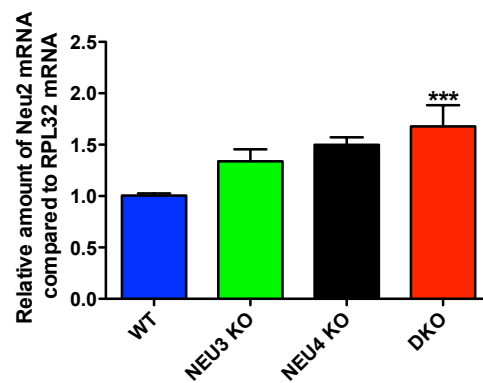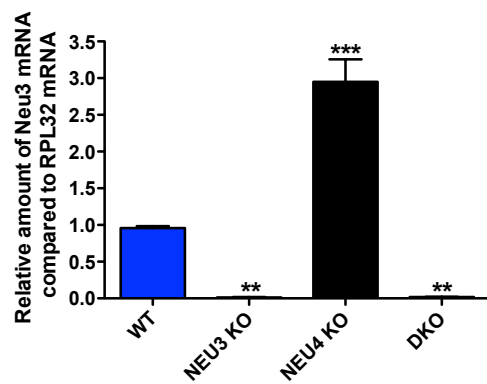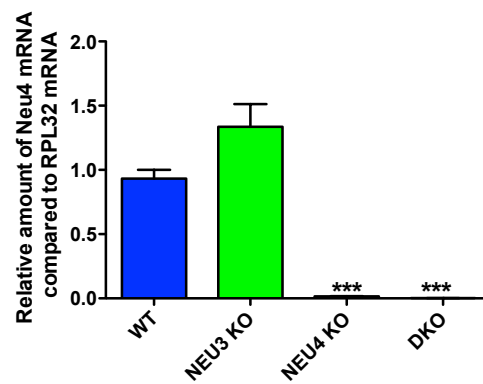

Figure S1.

Supplement: Figure S1 — Relative expression of neuraminidase mRNA in mouse brain tissues. Total mRNA was extracted from whole brains of 16 week-old WT, neu3 −/−, neu4 −/− and double-knockout neu3 −/−; neu4 −/− mice and analyzed for neu1, neu2, neu3 and neu4 expression by qRT-PCR The values were corrected for the level of control RPL32 mRNA. ** and *** -significantly different from WT (P<0.01 and P<0.001, respectively) by repeated measurements ANOVA. (PDF) [file pone.0106320.s001.pdf]

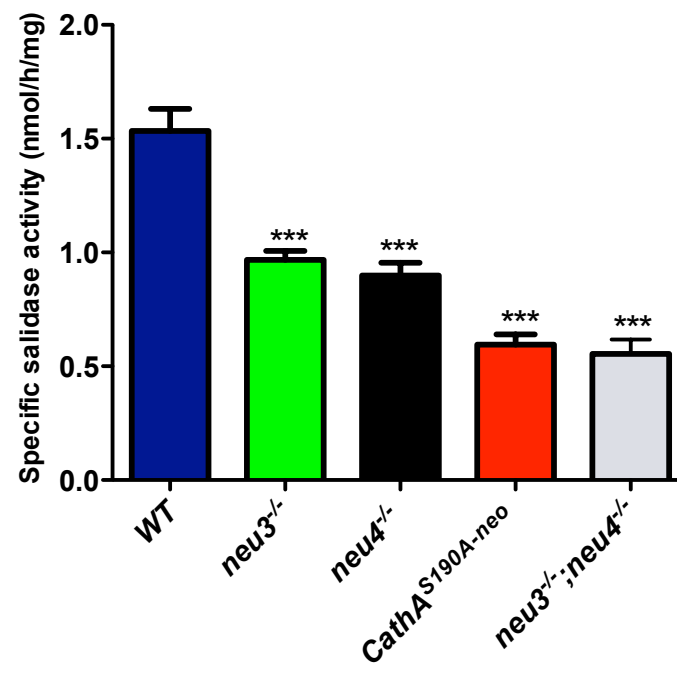

Figure S2.

Supplement: Figure S2 — 4MU-NANA neuraminidase activity in mouse brain tissues. Brain tissues of 16 week-old WT, neu3 −/−, neu4 −/−, CathAS190A-neo (neu1 KI) and double-knockout neu3 −/−; neu4 −/− mice and analyzed for neuraminidase activity against 4MU-NANA. Values are shown as means (±S.E). N-value for each genotype is as follows: WT and neu3 −/−; neu4 −/− n = 8, neu3 −/− and neu4 −/− n = 6; neu1 KI n = 4. *** -significantly different from WT (P<0.001) by repeated measurements ANOVA. (PDF) [file pone.0106320.s002.pdf]
